# USP36 inhibits apoptosis by deubiquitinating cIAP1 and survivin in colorectal cancer cells

**DOI:** 10.1016/j.jbc.2024.107463

**Published:** 2024-06-12

**Authors:** Bao Gao, Yuan Qiao, Shan Zhu, Ning Yang, Shan-Shan Zou, Yong-Jun Liu, Jingtao Chen

**Affiliations:** 1 Cancer Center, First Hospital of Jilin University, Changchun, Jilin, China; 2 Laboratory for Tumor Immunology, First Hospital of Jilin University, Changchun, Jilin, China

**Keywords:** apoptosis, B-cell lymphoma 2 (Bcl-2) family, colorectal cancer, deubiquitylation, NF-kappa B (NF-κB), polyubiquitin chain, posttranslational modification, protein degradation, protein–protein interaction, RNA interference

## Abstract

Chemotherapeutic agents for treating colorectal cancer (CRC) primarily induce apoptosis in tumor cells. The ubiquitin-proteasome system is critical for apoptosis regulation. Deubiquitinating enzymes (DUBs) remove ubiquitin from substrates to reverse ubiquitination. Although over 100 DUB members have been discovered, the biological functions of only a small proportion of DUBs have been characterized. Here, we aimed to systematically identify the DUBs that contribute to the development of CRC. Among the DUBs, ubiquitin-specific protease 36 (USP36) is upregulated in CRC. We showed that the knockdown of *USP36* induces intrinsic and extrinsic apoptosis. Through gene silencing and coimmunoprecipitation techniques, we identified survivin and cIAP1 as USP36 targets. Mechanistically, USP36 binds and removes lysine-11–linked ubiquitin chains from cIAP1 and lysine-48–linked ubiquitin chains from survivin to abolish protein degradation. Overexpression of *USP36* disrupts the formation of the XIAP–second mitochondria-derived activator of caspase complex and promotes receptor-interacting protein kinase 1 ubiquitination, validating USP36 as an inhibitor to intrinsic and extrinsic apoptosis through deubiquitinating survivin and cIAP1. Therefore, our results suggest that USP36 is involved in CRC progression and is a potential therapeutic target.

Colorectal cancer (CRC) accounts for approximately 10% of recently diagnosed cancers and cancer-related deaths (1). According to projections from the American Cancer Society, 153,020 new cases of CRC and 52,550 deaths from CRC will occur in the United States in 2023 (2). Low- and middle-income countries have witnessed an increase in the incidence of CRC. Additionally, the incidence of early-onset CRC, which occurs in individuals aged <50 years, has increased in high-income countries (3). Therefore, CRC remains a global public health concern.

Apoptosis evasion contributes to CRC pathogenesis. Fluoropyrimidines, oxaliplatin, and irinotecan form the backbone of treatment for stage III and certain stage II CRC (4); these treatments induce cancer cell death directly or indirectly through apoptotic pathways. Further understanding of the molecular mechanisms of apoptosis in CRC development may provide novel therapeutic targets.

Ubiquitination is vital for the apoptotic pathway. Ubiquitin is activated and conjugated to target proteins by the sequential actions of E1 (ubiquitin-activating enzyme), E2 (ubiquitin-conjugating enzyme), and E3 (ubiquitin ligase) (5). Ubiquitin chains linked *via* lysine at residue 48 or 11 target proteins to the 26S proteasome for degradation, and chains linked *via* lysine 63 are implicated in signaling events (6). Important regulators of apoptosis, including B-cell leukemia 2 (Bcl-2) and the inhibitor of apoptosis protein (IAP) family proteins, have been identified as ubiquitination substrates (7). Furthermore, some IAPs serve as E3 ligases and participate in ubiquitylation of apoptotic substrates (8).

Similar to other posttranslational modifications, ubiquitination is reversible. Deubiquitinating enzymes (DUBs) cleave ubiquitin from substrate proteins. Approximately, 100 DUBs are classified into six families, with ubiquitin-specific proteases (USPs) being the largest family (9). Genetic alterations in DUBs drive tumor progression. For example, *A20* is frequently inactivated by somatic mutations or deletions in B-cell lymphomas. Re-expressing wildtype (WT) *A20* downregulates nuclear factor kappa enhancer binding protein (NF-κB) signaling and induces apoptosis (10). Moreover, high expression levels of USP9X are correlated with increased myeloid cell leukemia sequence 1 (Mcl-1) expression in human follicular lymphomas and diffuse large B-cell lymphomas. *USP9X* knockdown enhances cell death caused by the Bcl-2 homology 3 mimetic ABT-737 (11). Disruption of *USP7* stabilizes and activates p53 (12), and inhibitors targeting USP7 can induce cell death in multiple tumors (13, 14, 15). Accordingly, drug design targeting DUBs may provide a new strategy for anticancer therapy, necessitating further investigation of the functions and mechanisms of DUBs in cancer.

USP36, a USP member, was initially isolated as a DUB enzyme from ovarian cancer cells. USP36 has recently attracted increasing attention in cancer studies from ovarian cancer (16) to breast cancer (17), lung cancer (18), hepatocellular carcinoma (19), esophageal squamous carcinoma (20), and glioblastoma (21). In these studies, USP36 was abnormally expressed in tumor tissues and related to poor prognosis, suggesting that *USP36* is an oncogene.

Here, we screened the Cancer Genome Atlas (TCGA) database and found that *USP36* expression is upregulated in CRC. We measured the effect of USP36 on apoptosis and the underlying molecular mechanisms. Our data confirm that USP36 is a novel regulator of cIAP1 and survivin and may represent a new therapeutic target for cancer treatment.

## Results

### USP36 expression is upregulated in CRC tissues and cells

**Figure 1:**
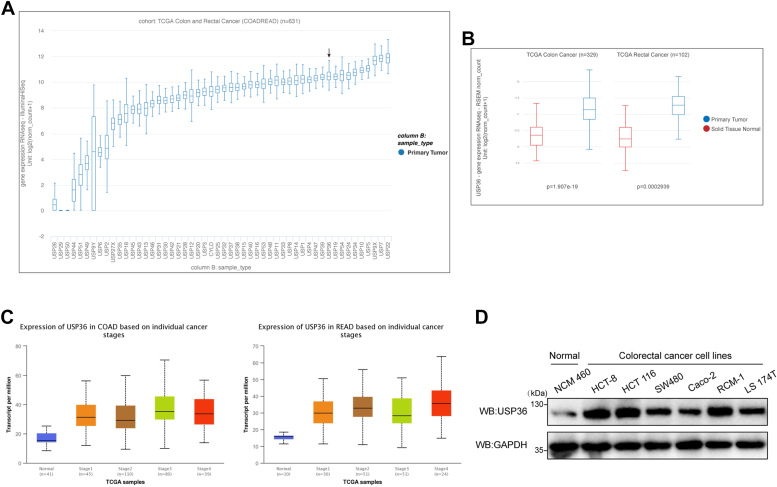
***USP36* expression is upregulated in CRC tissues and cells.***A*, USPs expression in tumor samples of patients with colorectal cancer from TCGA database. USP36 is marked. *B*, *USP36* expression levels in colon adenocarcinoma (COAD) and rectum adenocarcinoma (READ) tumors *versus* normal samples in TCGA are shown in the histogram. *C*, *USP36* expression in COAD and READ between normal and tumor tissues of different stages are presented in the histogram. *D*, Western blots of USP36 in CRC and control cell line NCM 460. CRC, colorectal cancer; TCGA, the Cancer Genome Atlas; USP36, ubiquitin-specific protease 36.

We first explored the mRNA levels of 52 USPs in colorectal carcinoma (Fig. 1*A*). Leading proteins, including USP7, USP9X, USP19 (22), and USP39 (23, 24), are known to be involved in tumor progression. To clarify the treatment potential of USPs, we compared their mRNA expression levels in tumor and normal tissue samples from patients in TCGA. Among the candidate USPs, *USP39* and *USP36* expression differed significantly (Fig. S1, *A* and *B*), consistent with observations in patients with colon and rectal cancer (Fig. 1*B*). Although USP39 functions have been extensively studied, the role of USP36 in colorectal carcinoma remains ambiguous. Additionally, *USP36* expression levels increased in colorectal tumor samples at all stages (Fig. 1*C*). The protein expression of *USP36* was measured in six different colorectal carcinoma cell lines and the control human normal colon epithelial cell line (NCM 460). USP36 protein expression was higher in colorectal carcinoma cell lines than in the control cell line, with the highest expression detected in HCT-8, HCT 116, and RCM-1 cell lines (Fig. 1*D*). These results indicate the importance of USP36 in CRC development and progression.

### USP36 downregulation can promote intrinsic and extrinsic apoptosis

**Figure 2:**
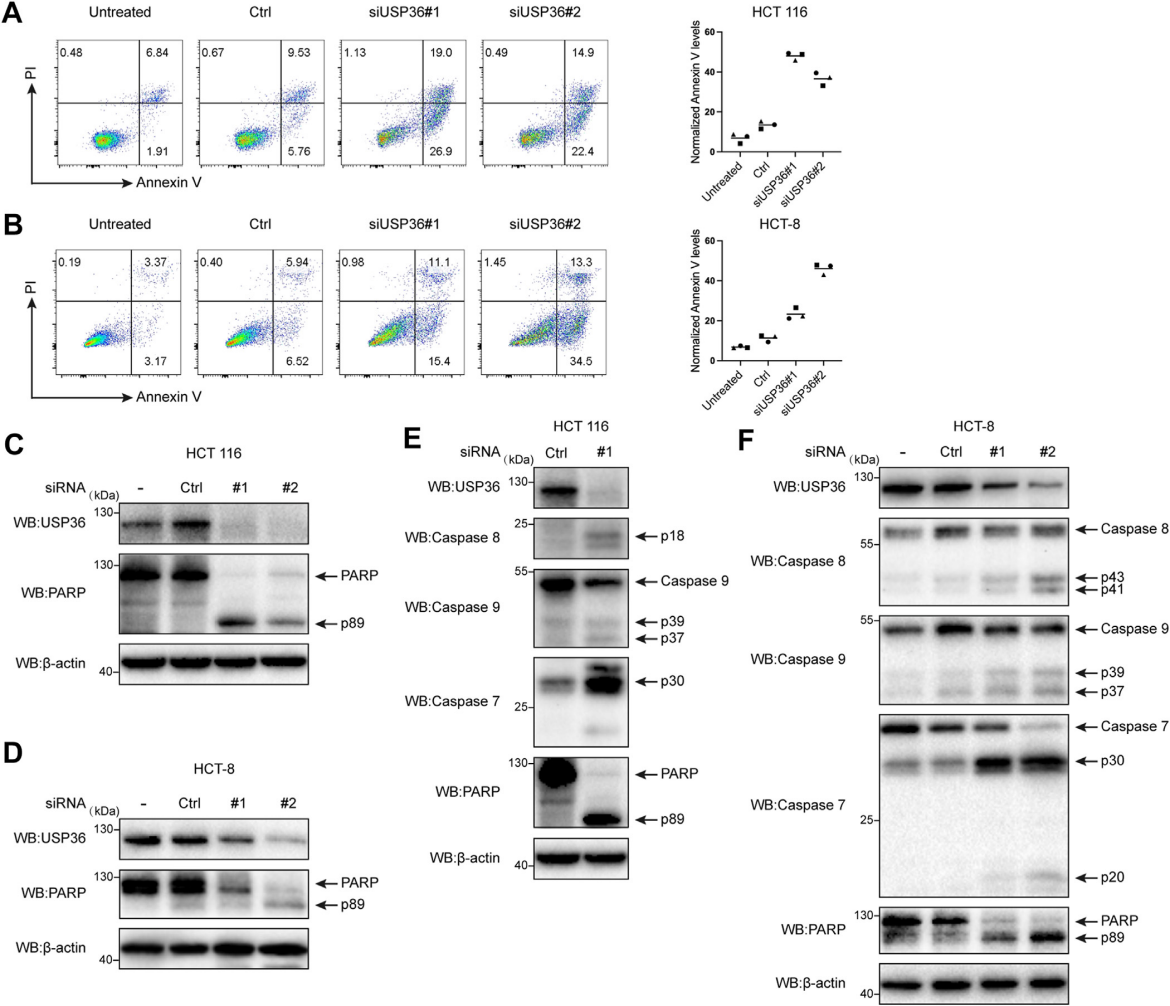
***USP36* downregulation can promote intrinsic and extrinsic apoptosis**. *A*, HCT 116 cells were analyzed for apoptosis following *USP36* knockdown based on Annexin V staining and nuclear propidium iodide (PI) staining content. *B*, HCT-8 cells were analyzed for apoptosis following *USP36* knockdown based on Annexin V staining and the content of nuclear PI staining. *C*, HCT 116 cells were untreated (−) or transfected with control siRNA (Ctrl), siUSP36#1 (#1), and siUSP36#2 (#2). Proteins were extracted and subjected to Western blot. *D*, HCT-8 cells were untreated (−) or transfected with control siRNA (Ctrl), siUSP36#1 (#1), and siUSP36#2 (#2). Proteins were extracted and subjected to Western blot. *E*, HCT 116 cells were transfected with control siRNA (Ctrl), siUSP36#1 (#1). Cell extracts were blotted with indicated antibodies. *F*, HCT-8 cells were untreated (−) or transfected with control siRNA (Ctrl), siUSP36#1 (#1), and siUSP36#2 (#2). Cell extracts were blotted with indicated antibodies. PARP, poly-ADP ribose polymerase; USP36, ubiquitin-specific protease 36.

We examined the role of USP36 in cell survival using two cancer cell lines, HCT 116 and HCT-8. Flow cytometry was performed to examine the effect of USP36 on cancer cell apoptosis. Annexin V staining revealed that small interfering (si)USP36#1 and siUSP36#2 RNA transfection significantly enhanced cell apoptosis compared to the untreated group (Fig. 2, *A* and *B*), which was further confirmed *via* Western blot analysis of poly-ADP ribose polymerase (PARP) cleavage (Fig. 2, *C* and *D*). Apoptosis is initiated by death receptor–induced extrinsic and mitochondria-mediated intrinsic pathways (25), which are characterized by the activation of cysteine-aspartic protease (caspase)-8 and caspase-9, respectively. Both pathways converge on downstream effector caspases, particularly caspase-3 and caspase-7, leading to the proteolysis of hundreds of cellular proteins (PARP and others). To explore the role of USP36 in apoptosis, we introduced siRNAs against *USP36* into two CRC (HCT 116 and HCT-8) cell lines (Fig. 2, *E* and *F*); caspase-7 was activated as determined by its cleavage into its active form after *USP36* knockdown. The cleavage of the endogenous caspase-7 substrate PARP showed the same pattern. Additionally, *USP36* knockdown cells showed higher caspase-8 and caspase-9 activation levels than control cells. These data suggest that *USP36* knockdown activated extrinsic and intrinsic apoptosis.

### USP36 downregulation diminishes the levels of several antiapoptotic proteins from the Bcl-2 and IAP families

**Figure 3:**
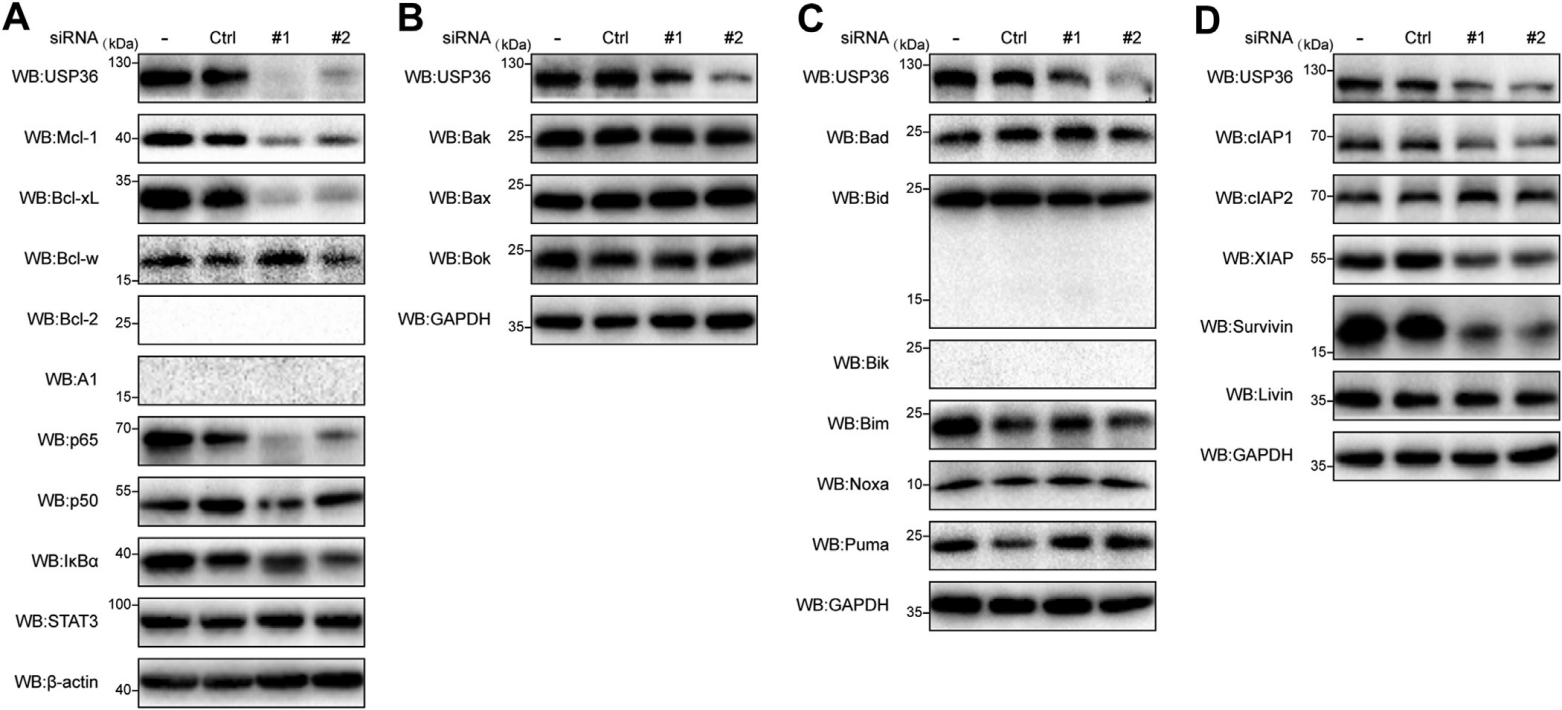
***USP36* downregulation diminishes the levels of several antiapoptotic proteins from the Bcl-2 and IAP families**. *A*, HCT 116 cells were untreated (−) or transfected with control siRNA (Ctrl), siUSP36#1 (#1), and siUSP36#2 (#2) for 72 h. Proteins were extracted and immunoblotted with antibodies against antiapoptotic proteins of the Bcl-2 family and transcription factors NF-κB and signal transducer and activator of transcription 3 (STAT3). *B*, HCT-8 cells were untreated (−) or transfected with control siRNA (Ctrl), siUSP36#1 (#1), and siUSP36#2 (#2) for 72 h. Proteins were extracted and immunoblotted with antibodies against proapoptotic pore-forming proteins of the Bcl-2 family. *C*, HCT-8 cells were untreated (−) or transfected with control siRNA (Ctrl), siUSP36#1 (#1), and siUSP36#2 (#2) for 72 h. Proteins were extracted and immunoblotted with antibodies against proapoptotic BH3-only proteins of the Bcl-2 family. *D*, HCT-8 cells were untreated (−) or transfected with control siRNA (Ctrl), siUSP36#1 (#1), and siUSP36#2 (#2) for 72 h. Proteins were extracted and immunoblotted with antibodies from IAP family proteins. Bcl-2, B-cell leukemia 2; Bcl-xL, Bcl-extra-large; Bid, Bcl-2 homology 3–interacting domain death agonist; IAP, inhibitor of apoptosis protein; Mcl-1, myeloid cell leukemia sequence 1; USP36, ubiquitin-specific protease 36.

According to previous reports, USP36 usually acts as a deubiquitinase to stabilize substrate proteins (26, 27). To investigate the molecular mechanisms underlying USP36 regulation of apoptosis in CRC cells, we analyzed a subset of proteins involved in apoptosis, specifically those with antiapoptotic functions. We focused on two major antiapoptotic protein families: Bcl-2 and IAP. Additionally, we identified the transcription factors that regulate Bcl-2 and IAP family members. Bcl-extra-large (xL) and Mcl-1 levels were markedly reduced after *USP36* silencing with siRNA (Fig. 3*A*). Additionally, Bcl-2 and A1 were absent in the Ctrl and siUSP36 groups, which has been previously reported to be absent in most solid tumors (Fig. S2) (28). NF-κB is an inducible transcription factor that controls the expression of antiapoptotic genes. It is predominantly composed of homodimeric and heterodimeric protein complexes containing p65 and p50 and binds to an inhibitor of kappa B to maintain its inactive form. After transfection with *USP36* siRNA, p65 expression was almost completely abrogated (Fig. 3*A*). Additionally, we detected Bcl-2 family members with proapoptotic functions, as some proteins could be upregulated during apoptosis. No evident upregulation was observed (Fig. 3, *B* and *C*). The Bcl-2 homology 3–interacting domain death agonist (Bid) can be cleaved by caspase-8 to generate a truncated (tBid) form, which enables crosstalk between the extrinsic and intrinsic apoptotic pathways. We did not detect a cleaved fragment (15 kDa) below the FL (22 kDa) of Bid (Fig. 3*C*). Finally, the levels of IAP family members, cIAP1, X-linked (X)IAP, and survivin, were significantly reduced after *USP36* silencing (Fig. 3*D*). These results identify Bcl-xL, Mcl-1, p65, cIAP1, XIAP, and survivin as USP36 substrates after gene knockdown. However, the protein that USP36 directly controls requires further elucidation.

### USP36 interacts with cIAP1 and survivin *in vivo*

**Figure 4:**
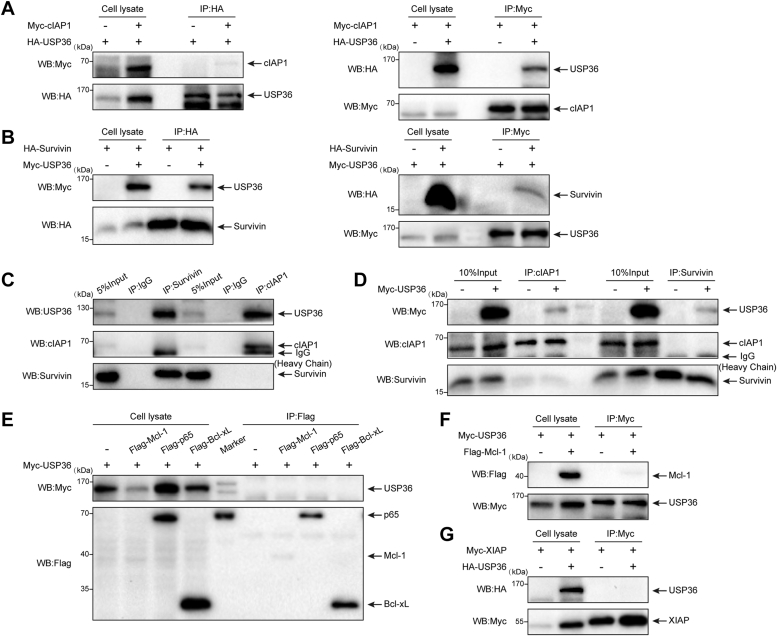
**USP36 interacts with cIAP1 and survivin *in vivo***. *A*, HEK-293 cells were transfected with Myc-cIAP1 and HA-USP36 individually or together. HA- or Myc-tagged proteins were immunoprecipitated from cell lysates and blotted with anti-HA and anti-Myc. *B*, HEK-293 cells were transfected with Myc-USP36 and HA-survivin individually or together. HA- or Myc-tagged proteins were immunoprecipitated from cell lysates and blotted with anti-HA and anti-Myc. *C*, HCT-8 cell lysates were immunoprecipitated with control IgG, anti-cIAP1, or anti-survivin antibody. Immunoprecipitates were analyzed *via* Western blot using indicated antibodies. *D*, HEK-293 cells were transfected with an empty vector or Myc-USP36, and protein interaction was assayed by immunoprecipitation with anti-cIAP1 or anti-survivin antibody and blotted with the indicated antibodies. *E*, HEK-293 cells were transiently transfected with Myc-USP36 alone or together with Flag-Bcl-xL, Flag-p65, or Flag-Mcl-1. Protein interaction was assayed by immunoprecipitation with anti-Flag magnetic beads and blotted with the indicated antibodies. *F*, HEK-293 cells were transiently transfected with Myc-USP36 alone or with Myc-USP36 and Flag-Mcl-1. Protein interaction was assayed using immunoprecipitation with anti-Myc beads and blotted with the indicated antibodies. *G*, HEK-293 cells were transiently transfected with Myc-XIAP alone or with Myc-XIAP and HA-USP36. Protein interaction was assayed using immunoprecipitation with anti-Myc magnetic beads and blotted with the indicated antibodies. HA, hemagglutinin; HEK, human embryonic kidney; IAP, inhibitor of apoptosis protein; Mcl-1, myeloid cell leukemia sequence 1; USP36, ubiquitin-specific protease 36.

Deubiquitinating enzymes regulate substrate proteins by binding to them (29). Immunoprecipitation analysis evaluated the interactions between USP36 and its potential targets *in vivo*. Ectopic Myc-cIAP1 was coimmunoprecipitated with hemagglutinin (HA)-USP36 using an anti-HA antibody when both plasmids were transfected (Fig. 4*A*, left panels). Additionally, HA-USP36 was coimmunoprecipitated with Myc-cIAP1 using an anti-Myc antibody when both plasmids were transfected (Fig. 4*A*, right panels). Moreover, endogenous USP36 was specifically immunoprecipitated by an anti-cIAP1 antibody rather than by control immunoglobulin G (IgG) (Fig. 4*C*, lane 6). As shown in Fig. 4*B*, ectopic Myc-USP36 was coimmunoprecipitated with HA-survivin using an anti-HA antibody, and ectopic HA-survivin was coimmunoprecipitated with Myc-USP36 using an anti-Myc antibody. Using the survivin-specific antibody, we observed that USP36 was present in the anti-survivin immunoprecipitates from the cell extracts of HCT-8, but not in the control IgG (Fig. 4*C*, lane 3). The findings that USP36 can interact with cIAP1 and survivin led us to examine whether these three components might form a protein complex. To test this, human embryonic kidney (HEK)-293 cells were transfected with Myc-tagged USP36, and anti-cIAP1 or anti-survivin immune complexes from the indicated lysates were immunoblotted for associated proteins (Fig. 4*D*). In agreement with previous results, Myc-USP36 was associated with cIAP1 and survivin. However, cIAP1 did not bind with survivin regardless of the expression of exogenous *USP36*. These data indicate that USP36 modulates cIAP1 and survivin separately. Finally, we determined the association of USP36 with Bcl-xL, p65, Mcl-1, and XIAP *via* coimmunoprecipitation in HEK-293 cells and observed no interactions (Fig. 4, *E*–*G*). These results suggest that USP36 regulates apoptosis *via* cIAP1 and survivin expression.

### Structural determinants of USP36–cIAP1 and USP36–survivin interactions

**Figure 5:**
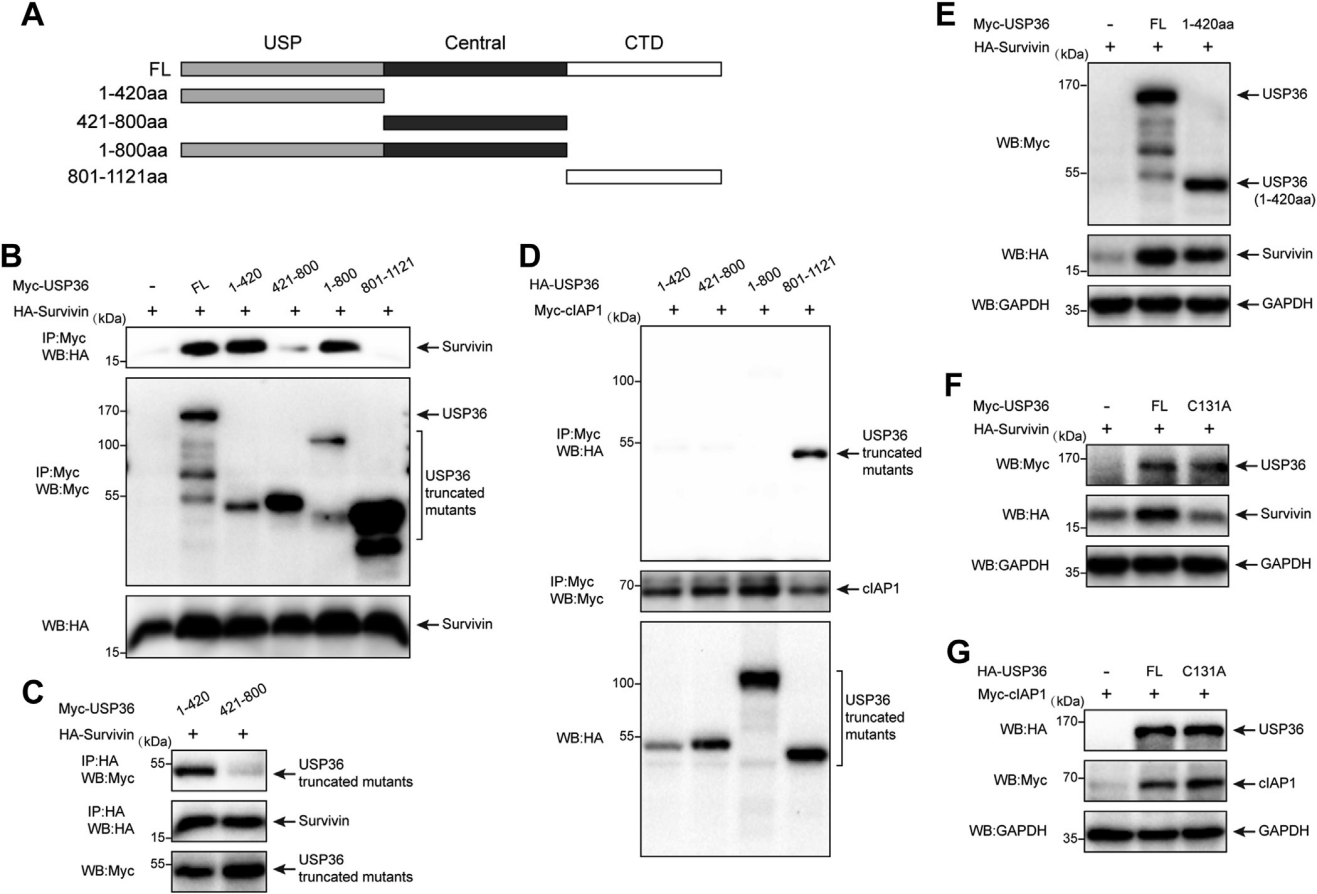
**Structural determinants of USP36-cIAP1 and USP36-survivin interactions**. *A*, schematic representation of the *USP36* and its deletion mutants used in this work. *B*, HA-survivin was expressed along with full-length USP36 (FL) or each of the indicated Myc-USP36 mutants. Cell lysates were immunoprecipitated with anti-Myc beads and blotted with anti-Myc and anti-HA antibodies. *C*, HA-survivin was expressed with 1 to 420 or 421 to 800 Myc-USP36 mutant. Cell lysates were immunoprecipitated with anti-HA beads and blotted with anti-HA and anti-Myc antibodies. *D*, Myc-cIAP1 was expressed along with each of the indicated HA-USP36 mutants. Cell lysates were immunoprecipitated with anti-Myc beads and blotted with anti-Myc and anti-HA antibodies. *E*, HEK-293 cells were transfected with survivin alone or together with Myc-tagged full-length USP36 or USP36 (1–420aa). Proteins were extracted and blotted with the indicated antibodies. *F*, HEK-293 cells were transfected with survivin alone or together with Myc-tagged full-length USP36 or USP36 (C131A). Proteins were extracted and blotted with the indicated antibodies. *G*, HEK-293 cells were transfected with cIAP1 alone or together with HA-tagged full-length USP36 or USP36 (C131A). Proteins were extracted and blotted with the indicated antibodies. HA, hemagglutinin; HEK, human embryonic kidney; IAP, inhibitor of apoptosis protein; USP36, ubiquitin-specific protease 36.

To determine the domains responsible for interacting with survivin and cIAP1, we constructed a panel of Myc- or HA-tagged *USP36* deletion mutants (Fig. 5*A*). USP36 comprises N-terminal USP [1–420 amino acids (aa)], central (421–800 aa), and C-terminal domains (801–1121 aa) (30). The 1 to 800 aa segment contains regions of the N-terminal and central domains. We cotransfected full-length (FL) USP36 or its deletion mutants with HA-survivin into HEK-293 cells and used an anti-Myc antibody to perform immunoprecipitation assays (Fig. 5*B*). Survivin specifically coimmunoprecipitated with mutants containing the N-terminal USP domain (lanes 2, 3, and 5) rather than with those without the domain. Additionally, we observed that the 421 to 800 aa domain of USP36 might interact with survivin. To rule out this possibility, HA-survivin and the 1 to 420 or 421 to 800 aa domains of USP36 were cointroduced into HEK-293 cells, and the cell lysates were immunoprecipitated with anti-HA antibodies, followed by blotting with anti-HA and anti-Myc antibodies (Fig. 5*C*). These results confirm that the 1 to 420 aa region of USP36 is necessary for binding to survivin. Immunoprecipitation experiments using cells transfected with various HA-USP36 domains showed that Myc-cIAP1 is selectively bound to the C-terminal domain (801–1121 aa) of USP36 (Fig. 5*D*, lane 4).

To address the role of the N-terminal of USP36 on survivin protein levels, we cotransfected HEK-293 cells with indicated plasmids (Fig. 5*E*). The expression of the 1 to 420 aa domain induced a similar increase in survivin protein levels compared to that of FL-USP36. The use of FL USP36 with catalytic residue Cys 131 mutated to Ala within the 1 to 420aa domain (C131A) did not increase survivin levels (Fig. 5*F*), further indicating that the 1 to 420 aa domain is required for stabilizing survivin, and the function is catalytic dependent. Notably, the C131A mutant, which has a functional 801 to 1121 domain, retained the ability to increase the levels of cIAP1 (Fig. 5*G*, lane 3), thereby indicating that the binding of USP36 is essential for cIAP1 stabilization.

### USP36 promotes survivin and cIAP1 stability by inhibiting ubiquitination

**Figure 6:**
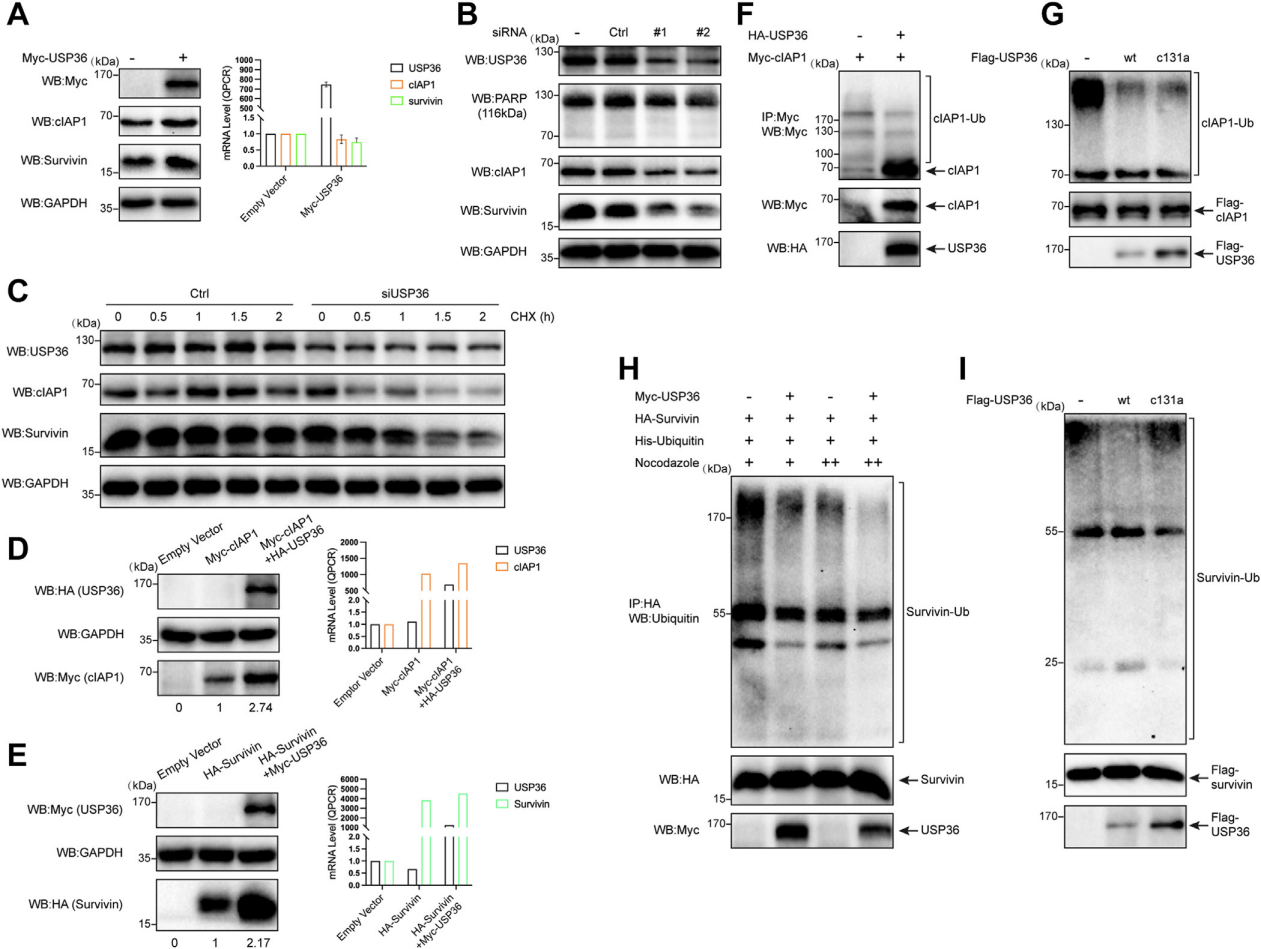
**USP36 promotes survivin and cIAP1 stability by inhibiting ubiquitination.***A*, HEK-293 cells were transfected with an empty vector or Myc-USP36 for 48 h. Cell lysates were harvested and analyzed for protein and mRNA levels of endogenous cIAP1 and survivin. Error bars denote s.e.m. (n = 3). *B*, HEK-293 cells were transfected with control or USP36-specific siRNAs. Western blot analysis of the protein levels using USP36, PARP, cIAP1, survivin, and GAPDH-specific antibodies. *C*, HCT 116 cells transfected with control or USP36 siRNA were treated with 20 μg/ml cycloheximide (CHX) for the indicated periods of time and analyzed *via* Western blot. *D*, HCT 116 cells were transfected with empty vector only, myc-cIAP1, or myc-cIAP1 plus HA-USP36. Cell lysates were subjected to Western blotting (*left panel*) or qRT-PCR analysis (*right panel*). *E*, HCT 116 cells were transfected with empty vector only, HA-survivin, or HA-survivin plus Myc-USP36. Cell lysates were subjected to Western blotting (*left panel*) or qRT-PCR analysis (*right panel*). *F*, Myc-cIAP1 was transfected into HEK-293 cells together with control or HA-USP36. Myc-tagged proteins were immunoprecipitated from cell lysates and blotted with the Myc monoclonal antibody. *G*, HEK-293 cells were transfected with Flag-cIAP1. After 24 h, the cells were treated with MG-132 (20 μM) for 1 h before being harvested. Ubiquitinated Flag-cIAP1 protein was purified with anti-Flag magnetic beads and eluted with 3×Flag peptide, followed by incubation with purified USP36 WT or USP36 C131A. Reaction mixtures were blotted with the anti-ubiquitin antibody. *H*, HEK-293 cells were cotransfected with the indicated plasmids. Cells were treated with nocodazole 400 nM (lanes 1 and 2) or 0.4 μg/ml (lanes 3 and 4) for 16 h before harvesting. Proteins were extracted, immunoprecipitated with HA beads, and blotted with the anti-ubiquitin (Ub) antibody. *I*, purified ubiquitinated survivin protein was incubated with purified proteins of either WT USP36 (lane 2) or USP36 C131A (lane 3) at 30 °C for 1 h, and the mixtures were blotted with the anti-ubiquitin antibody. HA, hemagglutinin; HEK, human embryonic kidney; IAP, inhibitor of apoptosis protein; USP36, ubiquitin-specific protease 36.

To assess whether USP36 modulates the endogenous levels of cIAP1 and survivin proteins, we performed transient transfection in HEK-293 cells. Overexpression of *USP36* increased the protein levels of endogenous cIAP1 and survivin in HEK-293 cells (Fig. 6*A*), whereas no discernible increase in the mRNA levels was observed. To exclude the possibility that reduction of cIAP1 and survivin when silencing *USP36* was the result of apoptosis, we performed knockdown assays in HEK-293 cells. As shown in Fig. 6*B*, the knockdown of *USP36* resulted in no enhancement in apoptosis; however, it still caused the downregulation of cIAP1 and survivin. To demonstrate that USP36 affects cIAP1 and survivin stability, we treated control cells or cells transfecting *USP36* siRNA with cycloheximide and examined protein half-life. cIAP1 and survivin stability decreased when *USP36* was knocked down (Fig. 6*C*). In HCT 116 cells, the overexpression of *USP36* also increased the protein levels of cIAP1 (Fig. 6*D*, left panels) and survivin (Fig. 6*E*, left panels), with a mild effect on the mRNA levels (Fig. 6, *D* and *E*, right panels). These results indicate that the regulation of cIAP1 and survivin stability by USP36 is not cell-type-specific and occurs through a posttranslational mechanism.

Considering the role of USP36 in stabilizing survivin and cIAP1, we examined whether USP36 acts as a deubiquitinase to cIAP1 and survivin. cIAP1 can catalyze its own ubiquitination and undergo degradation (31). We observed high levels of ubiquitinated cIAP1 in cells transfected with cIAP1 alone (Fig. 6*F*, lane 1). Overexpression of *USP36* resulted in a reduction in cIAP1 polyubiquitination, and thus, the protein levels of cIAP1 were increased (Fig. 6*F*, lane 2). To confirm the deubiquitinase activity of USP36 *in vitro*, we purified ubiquitinated cIAP1 from HEK-293 cells. The polyubiquitinated cIAP1 was incubated with purified Flag-USP36 (WT or C131A), followed by immunoblot. WT USP36 reduced cIAP1 ubiquitination (Fig. 6*G*, lane 2). Notably, C131A of USP36 also inhibited cIAP1 ubiquitination (Fig. 6*G*, lane 3), albeit to a lesser extent compared to WT USP36. Subsequently, we studied the effects of USP36 on survivin ubiquitination. Nocodazole is used to increase the percentage of mitotic cells and obtain high survivin ubiquitination levels (32). USP36 expression reduced the ubiquitin linkage on survivin induced by different nocodazole concentrations (Fig. 6*H*, lanes 2 and 4). In *in vitro* assays, WT USP36 effectively removed ubiquitin chains from survivin (Fig. 6*I*, lane 2), and USP36 C131A exhibited no DUB activity on survivin (Fig. 6*I*, lane 3). These data suggest that USP36 can act as a deubiquitinase of both cIAP1 and survivin to regulate protein stability. However, the catalytic domain of USP36 in regulating cIAP1 deubiquitination requires further investigation.

### USP36 disassembles K11-linked polyubiquitin chains from cIAP1 and K48-linked polyubiquitin chains from survivin

**Figure 7:**
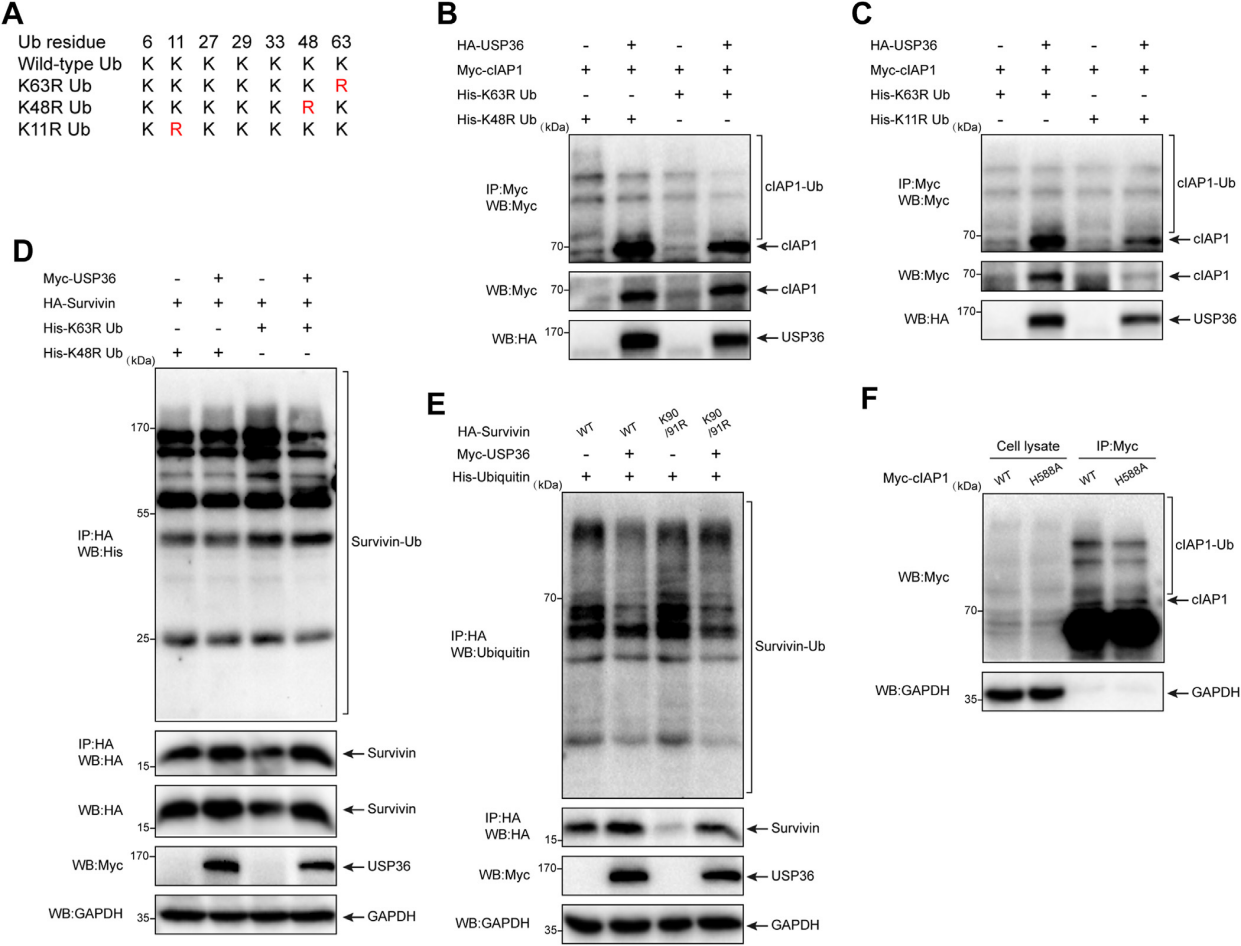
**USP36 disassembles K11-linked polyubiquitin chains from cIAP1 and K48-linked polyubiquitin chains from survivin**. *A*, schematic diagram of ubiquitin mutants. *B*, HEK-293 cells were cotransfected with the indicated plasmids. After 20 h, the cells were treated with 20 μM MG-132 for 4 hours. Proteins were extracted, immunoprecipitated with Myc beads, and blotted with anti-Myc antibody. *C*, HEK-293 cells were cotransfected with the indicated plasmids. After 20 h, the cells were treated with 20 μM MG-132 for 4 h. Proteins were extracted, immunoprecipitated with Myc beads and blotted with anti-Myc antibody. *D*, HEK-293 cells were cotransfected with the indicated plasmids. Cells were treated with 0.4 μg/ml nocodazole for 16 h before harvesting. Proteins were extracted, immunoprecipitated with HA beads, and then analyzed for ubiquitination levels by Western blotting with an anti-histone (His) antibody. *E*, wildtype (WT) or K90R/K91R mutant survivin was cotransfected with ubiquitin (lanes 1 and 3) or in combination with Myc-USP36 (lanes 2 and 4). Proteins were extracted, immunoprecipitated with HA beads, and then analyzed for ubiquitination levels using Western blotting with the anti-ubiquitin antibody. *F*, HEK-293 cells were transfected with wildtype cIAP1 (lanes 1 and 3) or H588A mutant cIAP1 (lanes 2 and 4). Proteins were extracted, immunoprecipitated with Myc beads, and blotted with the Myc antibody. HA, hemagglutinin; HEK, human embryonic kidney; IAP, inhibitor of apoptosis protein; USP36, ubiquitin-specific protease 36.

Given that USP36 mediates the deubiquitination of cIAP1 and survivin, we tested the ability of USP36 to cleave different types of polyubiquitin chains. We used the dominant-negative ubiquitin mutants K63R, K48R, and K11R, in which lysines 63, 48, and 11 were substituted with arginine (Fig. 7*A*). The conjugation of K48R or K63R ubiquitin to cIAP1 was markedly reduced in cells cotransfected with *USP36* (Fig. 7*B*, lanes 2 and 4). However, USP36 did not affect the K11R ubiquitin conjugation (Fig. 7*C*, lane 4). These findings confirm that cIAP1 can mediate K11-linked polyubiquitination independently (33) and suggest that USP36 affects cIAP1 with ubiquitin conjugation through lysine 11. Compared to K63R linkage ubiquitin (Fig. 7*D*, lane 3), K48R disrupted the targeting of survivin to degradation (Fig. 7*D*, lane 1), and USP36 showed no effect on the ubiquitination of survivin conjugated with K48R ubiquitin chains (Fig. 7*D*, lane 2). Thus, USP36 deubiquitinates survivin with selectivity for K48-linked ubiquitin chains.

Furthermore, we investigated which sites of survivin and cIAP1 are responsible for ubiquitin ligation and protein degradation. A double lysine mutant K90R/K91R has been reported to be stable against E3 ligase-mediated degradation of survivin (34). However, as shown in Fig. 7*E* (lane 3), the mutant survivin could also be ubiquitinated, and the expression level was considerably lower than that in the WT (Fig. 7*E*, lane 1). The expression of *USP36* still blocked the ubiquitination and degradation of survivin (Fig. 7*E*, lane 4). cIAP1 can promote polyubiquitination on itself; His^588^ residue within cIAP1 has proven critical for its E3-ligase activity, and the H588A mutant exhibits a higher expression than the WT cIAP1 (35). We detected that the ubiquitination of H588A mutant cIAP1 was attenuated; however, the protein levels remained the same as the WT (Fig. 7*F*, lane 4).

### USP36 inhibits apoptosis *via* the stabilization of survivin and cIAP1

**Figure 8:**
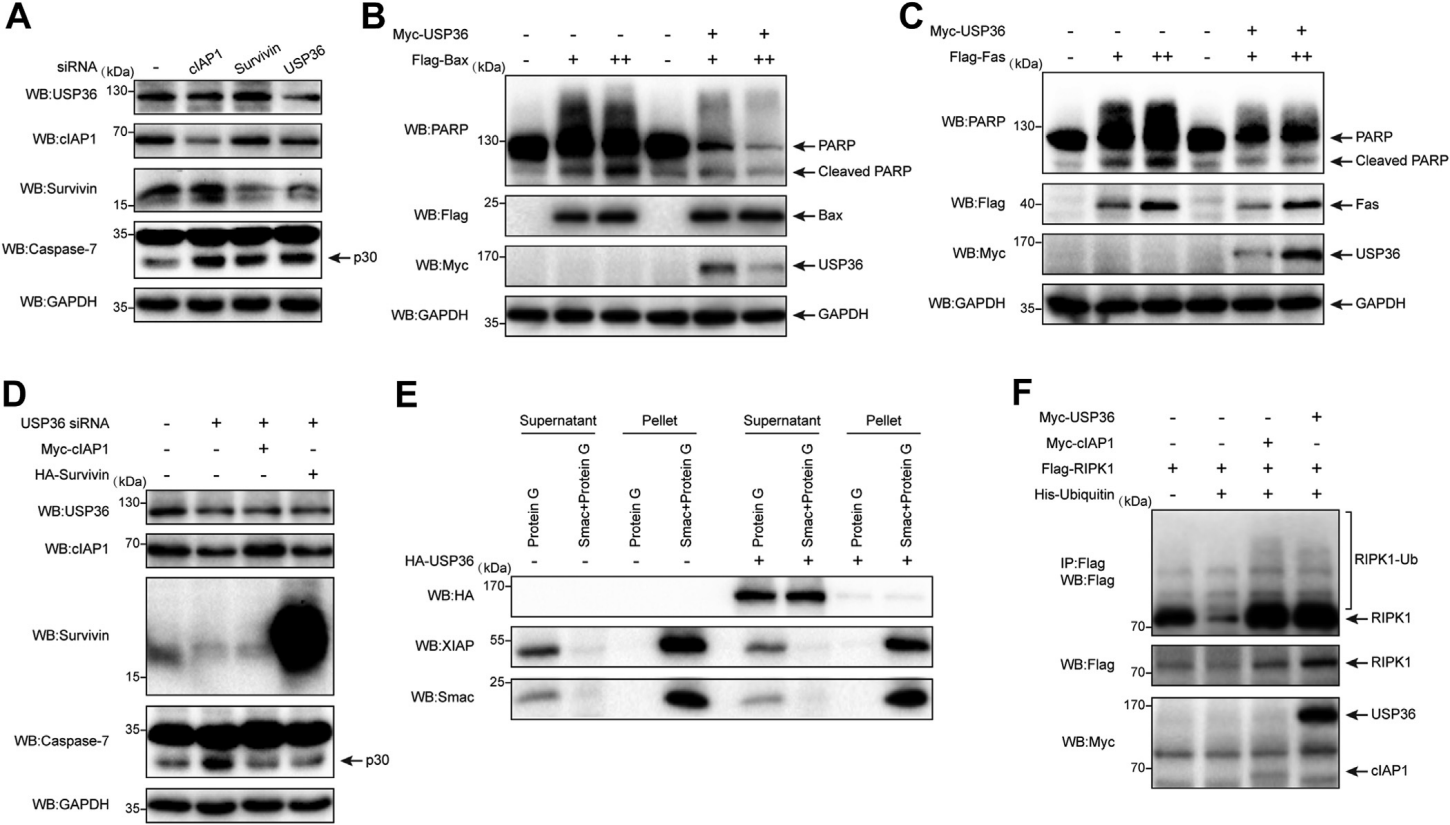
**USP36 inhibits apoptosis *via* the stabilization of survivin and cIAP1.***A*, HCT 116 cells were transfected with siRNA against the genes encoding cIAP1, survivin, or USP36 for 72 h. Proteins were extracted and subjected to Western blotting. Cell death was determined by cleavage of caspase-7. *B*, HCT 116 cells were transfected with increasing amounts of Flag-Bax individually or together with Myc-USP36. Cleavage of PARP indicated apoptosis in protein extracts. *C*, HCT 116 cells were transfected with increasing amounts of Flag-Fas individually or together with Myc-USP36. Cleavage of PARP indicated apoptosis in protein extracts. *D*, HCT 116 cells were transfected with USP36 siRNA alone (lane 2), with USP36 siRNA and Myc-cIAP1 together (lane 3), or with USP36 siRNA and HA-survivin together (lane 4). Cell death was determined by cleavage of caspase-7. *E*, HEK-293 cells were untreated (−) or transfected with HA-USP36. The uncoupled protein G and Smac-coupled protein G beads were collected after incubating with the cell lysates. Protein bound to the beads was eluted (Pellet). Supernatants from cell lysates treated with uncoupled protein G or Smac-coupled protein G beads, and pellets were analyzed using SDS-PAGE. *F*, HEK-293 cells were cotransfected with the indicated plasmids. Cells were harvested after 24 h. Proteins were extracted, immunoprecipitated with Flag beads, and blotted with the anti-Flag antibody. HA, hemagglutinin; HEK, human embryonic kidney; IAP, inhibitor of apoptosis protein; PARP, poly-ADP ribose polymerase; RIPK1, receptor-interacting protein kinase 1; Smac, second mitochondria-derived activator of caspase; USP36, ubiquitin-specific protease 36.

To further test whether USP36 has an antiapoptotic function through stabilizing cIAP1 and survivin, the genes encoding cIAP1 and survivin were individually knocked down. Downregulation of cIAP1 or survivin induced elevated levels of caspase-7 activation (Fig. 8*A*, lanes 2 and 3), which is a marker of undergoing apoptosis. Although less efficient than siRNA treatments, *USP36* knockdown reduced cIAP1 and survivin levels and induced apoptosis (Fig. 8*A*, lane 4). To determine whether USP36 inhibited apoptosis, transient transfection experiments were performed in HCT 116 cells using overexpression of Bax or Fas plasmids as stimuli for inducing intrinsic and extrinsic apoptosis, respectively. Transfection with different amounts of Flag-Bax plasmid induced apoptosis in a dose-dependent manner, as indicated by PARP cleavage (Fig. 8*B*, lanes 2 and 3). However, cell death was reduced when the USP36-encoding plasmid was cotransfected with Bax (Fig. 8*B*, lanes 5 and 6). Moreover, the expression of *USP36* suppressed cell death induced by Fas (Fig. 8*C*, lanes 5 and 6). Subsequently, Myc-cIAP1 and HA-survivin were individually transfected into the *USP36*-knockdown cells (Fig. 8*D*, lanes 3 and 4). Both cIAP1 and survivin prevented the cleavage of caspase-7 caused by *USP36* knockdown. These results suggest that USP36 suppresses apoptosis induced by diverse apoptotic stimuli by stabilizing cIAP1 and survivin.

To better understand the mechanisms of USP36 in regulating intrinsic and extrinsic apoptotic pathways, we tested the effect of USP36 on XIAP ubiquitination. Previous reports suggested that survivin interacts with XIAP and promotes its stability against ubiquitination and degradation, thereby inhibiting apoptosis (36). Our results support this hypothesis (Fig. S3*A*, lane 2), and we found that the interaction tended to promote, and not suppress, XIAP ubiquitination. Similar to survivin, USP36 could polyubiquitinate XIAP when overexpressed (Fig. S3*B*, lane 2). The ubiquitination of XIAP is critical for its antiapoptotic function (37). XIAP lacking the RING domain exhibits reduced levels of polyubiquitination and impaired antiapoptotic function compared to FL XIAP (38). In a previous study, survivin was found to compete with second mitochondria-derived activator of caspase (Smac) for binding to XIAP (39). As shown in Fig. 8*E* (lane 9 *versus* lane 4), *USP36* overexpression alleviated the formation of XIAP-Smac complex, akin to the effect observed with survivin, which could enhance the inhibition ability of caspase-9 on intrinsic apoptosis. Moreover, the receptor-interacting protein kinase 1 (RIPK1) is an important substrate of cIAP1. cIAP1 can promote RIPK1 ubiquitination to prevent the formation of Complex II with Fas-associated death domain and pro-caspase-8, thereby switching RIPK1 from a proapoptotic adaptor to a prosurvival scaffold protein (40). USP36 stabilizes cIAP1 expression, which led us to test the possibility that USP36 also modulates RIPK1 ubiquitination. As shown in Fig. 8*F*, RIPK1 ubiquitination was enhanced by cIAP1 (lane 3) and USP36 (lane 4). Therefore, we conclude that USP36 plays a crucial role in extrinsic and intrinsic apoptosis by stabilizing cIAP1 and survivin.

## Discussion

The switch “on and off” of apoptosis is a fine-tuning process determined by the ratio of proapoptotic and antiapoptotic proteins. In most cells, cell death can be blocked by the abnormal expression of antiapoptotic proteins. IAP proteins are a major family of antiapoptotic molecules that prevent apoptosis by interfering with caspase activation. Eight members of the IAP gene family have been identified, including the most studied cIAP1, cIAP2, XIAP, and survivin (41). The unifying feature of IAP proteins is the baculoviral IAP repeat domain, which is required for IAP-mediated protein–protein interactions and the inhibition of apoptosis. Additionally, XIAP, cIAP1, and cIAP2 contain a RING domain, which allows them to serve as E3 ubiquitin ligases. We established that USP36 acts as a DUB for cIAP1 and survivin. As an interacting protein, USP36 cleaves K11- and K48-linked polyubiquitin chains from cIAP1 and survivin, respectively, thus protecting them from degradation and inhibiting apoptosis in CRC.

**Figure 9:**
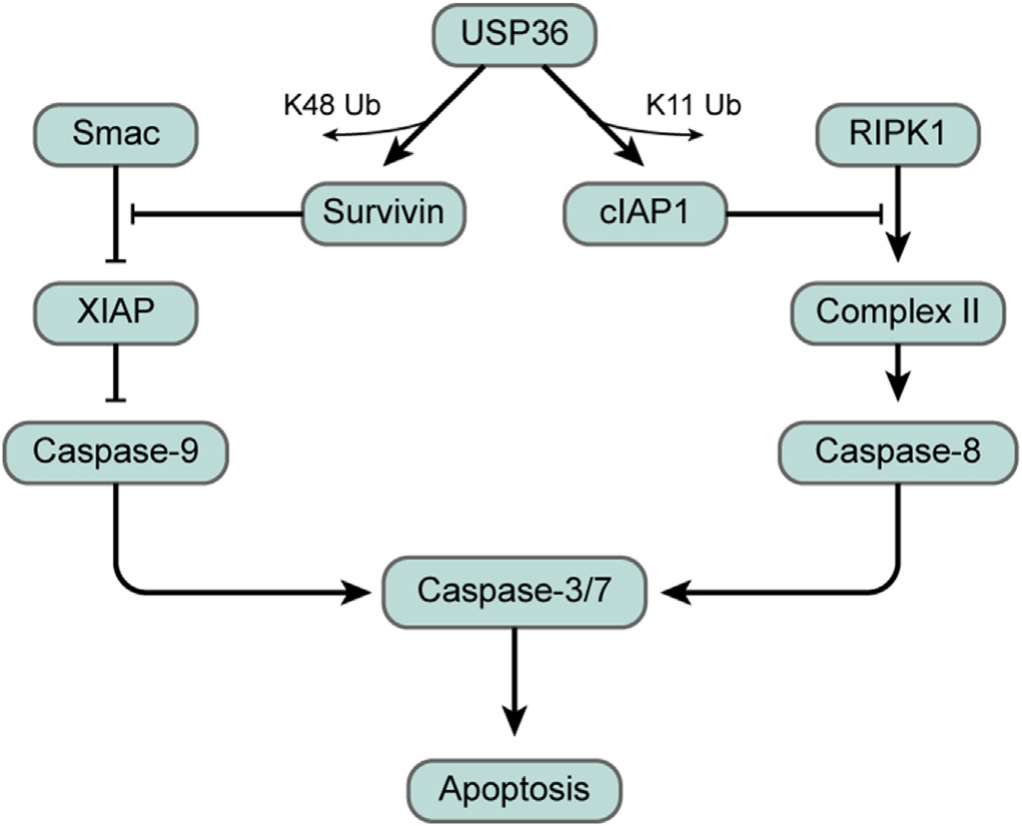
**A model for the regulation of apoptosis by USP36 *via* survivin and cIAP1**. RIPK1, receptor-interacting protein kinase 1; Smac, second mitochondria-derived activator of caspase; USP36, ubiquitin-specific protease 36.

High cIAP1 expression levels have been reported in colon cancer and have been associated with resistance to several anticancer drugs (42). In the absence of cIAP1, RIPK1 is not ubiquitinated, and nonubiquitinated RIPK1 assembles with Fas-associated death domain and pro-caspase-8 to form Complex II. It activates downstream caspases, thus inducing the extrinsic pathway of apoptosis (40). In this study, USP36 disassembles K11-linked polyubiquitin chains from cIAP1 and prevents its degradation. cIAP1-mediated ubiquitylation of RIPK1 blocked the formation of Complex II, leading to cell survival (Fig. 9). In addition, cIAP1 is a critical E3 ligase responsible for the proper functioning of the NF-κB pathway, which modulates the transcription of multiple prosurvival genes such as *BCL2L1* (Bcl-xL) (43). In our study, we observed reduced expression of p65 accompanied with decreased Bcl-xL levels. This event might also contribute to CRC cell apoptosis, as *BCL2L1* silencing has been shown to induce apoptosis in CRC (44).

In a previous study, cIAP1 expression was consistently significantly weaker than that of XIAP, suggesting that cIAP1 is an unstable protein (45). The deubiquitinating enzyme otubain 1 (46) and USP19 (47) can act as deubiquitinases to stabilize cIAP1, and otubain 1 has been implicated in removing K48-linked polyubiquitin chains from cIAP1. Upon external stimulation, cIAP1 commonly undergoes K11- and K63-ubiquitination (46), and K11-linked ubiquitin chains are characterized as degradative signals. In this study, we demonstrated that USP36 could reverse the K11-ubiquitination of cIAP1. However, whether this action relies on enzymatic activity remains controversial, as the USP36^C131A^ mutant retains the ability to stabilize cIAP1. In *in vitro* assays, the USP36^C131A^ mutant can disassemble ubiquitin chains on cIAP1. This suggests that another enzymatic domain of USP36 may exist, which cooperates with the C131 site to facilitate the deubiquitination of cIAP1. Moreover, we identified the C-terminal domain of USP36, which is considered instrumental for nucleoli localization (26), as the major region that interacts with cIAP1 (Fig. 5*D*). It cannot be excluded that USP36 prevents cIAP1 from relocating to the cytoplasm or nucleoplasm, thereby protecting it from ubiquitination and degradation by some unknown E3 ligases.

Survivin, the smallest member of the IAP family, is overexpressed in fetal organs and human tumors (48), especially in colon tumors. In patients with CRC, survivin is associated with reduced tumor cell apoptosis and poor prognosis (49). Although the effect of survivin in inhibiting apoptosis has been validated, the mechanism remains unclear. Several groups have suggested interactions between survivin and caspases, including caspase-3, caspase-7 (50), and caspase-9 (51). However, these interactions were weak or could not be confirmed in the cellular context. Other studies have suggested crosstalk between survivin and other IAPs. Our data confirmed this observation, which shows that survivin interacts with XIAP. Survivin might compete with Smac for binding to XIAP, increasing XIAP ubiquitination, thus blocking the activity of caspase-9 to inhibit intrinsic apoptosis (Fig. 9). USP36 acts as a deubiquitinase to prevent degradation of survivin, thus attenuating Smac–XIAP interaction (Fig. 8*E*). The selective role of survivin in antagonizing mitochondrial-dependent apoptosis has also been demonstrated in transgenic animal experiments (52).

Despite its shared high sequence similarity with cIAP1, the expression of cIAP2 was not impaired by *USP36* knockdown in our study (Fig. 3*D*), which might be attributed to their different subcellular distribution. cIAP1 is a primarily nuclear protein, whereas cIAP2 can be found in the cytoplasm and nuclear (53). In addition, cIAP2 was expressed in slightly higher amounts when cIAP1 was reduced by *USP36* knockdown. This result was consistent with a previous study reporting that elevated levels of cIAP2 protein can be observed in *BIRC2* (cIAP1)-deficient mice (54), suggesting a difference in regulating cIAP1 and cIAP2 expression.

To date, only a few DUBs of survivin have been identified. hFAM (USP9X)-mediated K63 deubiquitination is required to dissociate survivin from centromeres and maintain proper mitosis (32). Our results suggest that USP36 serves as a DUB but prefers to cleave the K48 ubiquitin chains of survivin. The fate of survivin is similar to that of c-Myc, another confirmed USP36 substrate (17). They all interact with the N-terminal region of USP36, and the C131A mutant of USP36 abolishes c-Myc and survivin induction; however, the relationship between survivin and c-Myc remains unknown.

Several cIAP1 antagonists have been reported recently and have been shown to possess proapoptotic activity; however, survivin-inhibiting-based cancer therapy is still in its infancy. Although YM155, presently the leading compound anticipated to inhibit survivin expression, effectively induces cell death, and its mechanism of action may not be related to survivin inhibition. Our data demonstrated that USP36 stabilizes survivin in a catalytic-dependent manner. Therefore, a peptide including the C131 residue of the USP36 sequence might block the interaction between survivin and USP36. This peptide, coupled with protein transduction domains (54), could be formulated into a cell-permeable peptidomimetic to provide an anticancer agent.

Some oncogenes, such as *murine double minute 2*, *Myc*, *USP7*, and *baculoviral IAP repeat containing 5*, are critical for embryonic development. Deficiencies in these genes can result in embryonic lethality and sensitivity to apoptosis. *USP36* is also essential for embryo development (55). These genes are undetectable in most normal adult tissues. However, they are frequently observed in human cancers, suggesting disruption of a delicate balance caused by transcriptionally and posttranslationally modified proteins. Furthermore, USP36 can be polyubiquitinated and degraded (56); however, the E3 ligases that execute this action are unknown, necessitating further investigation. Moreover, studies should be conducted to identify the transcription factors that target USP36.

Overall, our findings indicated that USP36 regulates cIAP1 and survivin ubiquitin levels. USP36 may be an appropriate target for drug discovery owing to its sharp differential expression in CRC.

## Experimental procedures

### TCGA data analysis

Gene expression in the CRC cohort in TCGA was analyzed using the UCSC Xena browser (57). Boxplots showing *USP36* mRNA expression levels in different stages of colon and rectal cancer were generated from the UALCAN web portal (58).

### Plasmids, antibodies, and reagents

Plasmids expressing Myc-USP36 (WT, Cat# PPL01459-2a), Flag-Mcl-1 (Cat# PPL00450-2a), Flag-p65 (Cat# BC011603), Myc-XIAP (Cat# PPL00449-2a), and HA-survivin (Cat# PPL02132-2a) were purchased from the Public Protein/Plasmid Library. Plasmids expressing Flag-HA-USP36 (WT, Cat# 22579, RRID: Addgene_22579) and Myc-cIAP1 (Cat# 8311, RRID: Addgene_8311) were purchased from Addgene. Plasmids expressing Flag-RIPK1 (Cat# F122510) were purchased from Fenghui Biotechnology. Plasmids expressing Flag-Bcl-xL (Cat# P24695), Flag-Bax (Cat# P37963), Flag-Fas (Cat# G15096), Myc-cIAP1-H588A, HA-survivin-K90R/K91R, His-ubiquitin (WT Cat# P28355, K11R Cat# P45614, K48R Cat# P7778, and K63R Cat# P7779), and truncated variants of USP36 (Myc- and HA-tagged) were purchased from Miaoling Biology. Flag-cIAP1 and Flag-survivin plasmids were purchased from He Wu Biology. Moreover, Myc-USP36 and Flag-HA-USP36 were used as templates to create the catalytically inactive USP36 variant, C131A. We used antibodies against USP36 (Cat# 14783-1-AP, RRID: AB_2213357), glyceraldehyde-3-phosphate dehydrogenase (Cat# 10494-1-AP, RRID: AB_2263076), STAT3 (Cat# 10253-2-AP, RRID: AB_2302876, Proteintech), β-actin (Cat# HC201, TransGen), caspase-7 (Cat# 9494), caspase-8 (Cat# 9746), caspase-9 (Cat# 9504), PARP (Cat# 9532), Mcl-1 (Cat# 94296), Bcl-xL (Cat# 2764), Bcl-2 (Cat# 4223), A1 (Cat# 14093), Bcl-w (Cat# 2724), NF-κB p65 (Cat# 4764), NF-κB p50 (Cat# 13586), IκBα (Cat# 4812), Bcl-2 Antagonist/Killer (Bak) (Cat# 12105), Bax (Cat# 5023), Bcl-2 related ovarian killer (Bok) (Cat# 86875), Bcl-2 associated agonist of cell death (Cat# 9239), Bid (Cat# 2002), Bcl-2 interacting killer (Cat# 4592), Bcl-2 interacting mediator of cell death (Bim) (Cat# 2933), Noxa (Cat# 14766), p53 upregulated modulator of apoptosis (Puma) (Cat# 12450), cIAP1 (Cat# 7065), cIAP2 (Cat# 3130), XIAP (Cat# 2045), survivin (Cat# 2808), Livin (Cat# 5471), HA-Tag (Cat# 3724), Myc-Tag (Cat# 2276), Flag-Tag (Cat# 14793), His-Tag (Cat# 9991), Ubiquitin (Cat# 3936) (Cell Signaling Technology), and Smac (Cat# sc-393118) (Santa Cruz Biotechnology). 3×Flag Peptide (Cat# P9801) was purchased from Beyotime Biotechnology. Finally, MG132 (Cat# ab141003, Abcam), nocodazole (Cat# M1404; Sigma-Aldrich), cycloheximide (Cat# 2112; Cell Signaling Technology), fluorescein isothiocyanate (FITC) Annexin V, and propidium iodide (PI) staining solution (Cat# 556547, RRID: AB_2869082; BD Biosciences) were used.

### Cells and transfections

NCM 460, LS 174T, and HEK-293 cells were maintained in Dulbecco's Modified Eagle’s medium supplemented with 10% fetal bovine serum (FBS), and Caco-2 cells were maintained in Dulbecco's Modified Eagle’s medium supplemented with 20% FBS. HCT-8 and HCT 116 were maintained in Roswell Park Memorial Institute-1640 medium supplemented with 10% FBS. SW480 cells were maintained in Leibovitz's L-15 medium supplemented with 10% FBS at 37 °C in atmospheric air without CO_2_. RCM-1 cells were grown in a medium containing 45% Roswell Park Memorial Institute-1640, 45% F-12 Hams, and 10% FBS. HEK-293 and HCT 116 were transiently transfected with plasmids using Lipofectamine 2000 (Cat# 11668019; Invitrogen) or polyethylenimine 40,000 (Cat# 40816ES02; Yeasen), according to the manufacturer's protocol.

### RNA interference

*USP36* siRNA duplexes #1 (5′-GGA GAU CCG GCA AGC UGC GAA UAU U-3′) and #2 (5′-CAG AUU CUA AGA CGG UGA AGC UGA A-3′) were synthesized by Invitrogen. Ctrl siRNA duplexes (Cat# 140630) were purchased from Sigma-Aldrich. cIAP1-siRNA (Cat# sc-29848) and survivin-siRNA (Cat# sc-29499) were purchased from Santa Cruz. Transfection was performed using Lipofectamine RNAiMAX (Cat# 13778150, Invitrogen) or Lipofectamine 2000 following the manufacturer's protocol, and the cells were collected after 48 or 72 h for analysis.

### Analysis of apoptosis

Apoptotic cells were determined *via* FITC Annexin V and PI staining. Briefly, the cells were harvested, suspended in 1× binding buffer (BD Biosciences), and stained with Annexin V and PI for 15 min. Next, cells were subjected to flow cytometry to measure apoptosis. Cells that stained positive for FITC Annexin V and negative for PI were considered to be undergoing apoptosis and those that stained positive for both FITC Annexin V and PI were in the end stage of apoptosis.

### Western blot analysis and immunoprecipitations

Western blots were performed using whole cell extracts, separated on 10 to 12% sodium dodecyl sulfate polyacrylamide gel electrophoresis gels, and transferred to 0.45 μm polyvinylidene difluoride membranes (Cat# IPVH00010; Millipore). Blots were probed with the indicated antibodies. The HEK-293 cells were transfected with the indicated plasmids. After 24 h, the cells were lysed in cell lysis buffer (Cat# 9803; Cell Signaling Technology) supplemented with protease inhibitor cocktail (Cat# HY-K0010; MedChemExpress), and protein complexes were immunoprecipitated with Myc-Tag (Cat# 5698; Cell Signaling Technology), HA-Tag (Cat# 11846; Cell Signaling Technology), anti-Flag (M2) magnetic beads (Cat# M8823; Sigma-Aldrich), or the indicated antibodies conjugated to protein A agarose beads(Cat# 9863; Cell Signaling Technology) and protein A magnetic beads (Cat# 73778; Cell Signaling Technology) or protein G magnetic beads (Cat# 70024; Cell Signaling Technology). To minimize the interference produced by the denatured rabbit heavy chain, mouse anti-rabbit IgG (Cat# 45262; Cell Signaling Technology) was used as a secondary antibody; to minimize interference produced by the denatured mouse heavy chain, rabbit anti-mouse IgG (Cat# 58802; Cell Signaling Technology) was used as a secondary antibody. To show specific binding in survivin and cIAP1 immunoprecipitations, rabbit monoclonal antibody IgG isotype control (Cat# 3900) was used.

### *In vivo* deubiquitination assays

HEK-293 cells were transfected with the indicated plasmids and, in some cases, pretreated with 20 μM MG-132 to block protein degradation. Next, the cells were collected and lysed, and the lysates were cleared through centrifugation. Subsequently, Myc-Tag or HA-Tag magnetic beads were added. The mixture was incubated with rotation overnight at 4 °C. Afterwards, the beads were washed five times and dissociated in protein loading buffer (Cat# DL101–02; TransGen) by heating at 95 °C for 5 min, and the supernatant was loaded to an sodium dodecyl sulfate polyacrylamide gel electrophoresis gel for Western blot analysis.

### *In vitro* deubiquitination assays

Both the enzymes (Flag-USP36, Flag-USP36-C131A) and the substrates (Flag-cIAP1, Flag-survivin) were individually expressed in HEK-293 cells in 10-cm dishes and purified with anti-Flag M2 Magnetic Beads in cell lysis buffer. After extensive washing with tris-buffered saline, the proteins were eluted with 150 μg/ml 3×Flag peptides. For the deubiquitination assay reaction *in vitro*, the ubiquitinated cIAP1 or survivin protein was incubated with USP36 or its catalytic mutant in a deubiquitination buffer (59) at 30 °C for 1 h and then subjected to Western blotting using anti-ubiquitin antibodies.

### Quantitative reverse transcriptase-PCR

Total RNA was isolated from cells using EasyPure RNA Kit (Cat# ER101–01; Transgen), and the first-strand cDNA was synthesized with EasyScript First-Strand cDNA Synthesis SuperMix (Cat# AT301–03; Transgen). Quantitative reverse transcriptase-PCR was performed on an ABI StepOne Plus real-time PCR system (Applied Biosystems) using SYBR Green Mix (Cat# 491391400; Roche). Relative expression levels of each gene were normalized to β-actin as the endogenous control for all experiments. The primers used in this study were as follows: USP36-forward-primer: 5′-AGCACTTTTCCCCCAGAACTG-3′; USP36-reverse-primer: 5′-GGCTCCCAGATCTGCTGCTA-3′; cIAP1-forward-primer: 5′-TCCAGCCTTTCTCCAAACCC-3′; cIAP1-reverse-primer: 5′-ACCAGCTCTTGCCAATTCTGA-3′; survivin-forward-primer: 5′-CACCGCATCTCTACATTCAAGA-3′; survivin-reverse-primer: 5′-AAGTCTGGCTCGTTCTCAGTG-3′; β-actin-forward-primer: 5′-TCGTGCGTGACATTAAGGAG-3′; and β-actin-reverse-primer: 5′-ATGCCAGGGTACATGGTGGT-3′.

### Statistical analysis

Statistical analyses were performed using GraphPad Prism 7.0 (GraphPad Software). A two-sided Student’s *t* test was used to compare two groups, and one-way analysis of variance was used to test for differences between more groups. Statistical significance was set at *p* < 0.05.

## Data availability

The data generated in this study are available upon request from the corresponding author.

## Supporting information

This article contains supporting information.

## Conflict of interests

The authors declare that they have no conflicts of interest with the contents of this article.
