# Supplementary material for: USP36 inhibits apoptosis by deubiquitinating cIAP1 and survivin in colorectal cancer cells
Source: J Biol Chem. 2024 Jun 12;300(7):107463. doi: 10.1016/j.jbc.2024.107463 (PMC11268115; doi:10.1016/j.jbc.2024.107463)
Supplement: Supporting information [file mmc1.pdf]

Supporting Information

Title: USP36 inhibits apoptosis by deubiquitinating cIAP1 and survivin in colorectal cancer cells

Authors: Bao Gao[1,2], Yuan Qiao[2], Shan Zhu[1,2], Ning Yang[2], Shan-Shan Zou[2], Yong-Jun Liu[2]*, and Jingtao Chen[1,2]*

Material included:

1. Figure S1
2. Figure S2
3. Figure S3

A

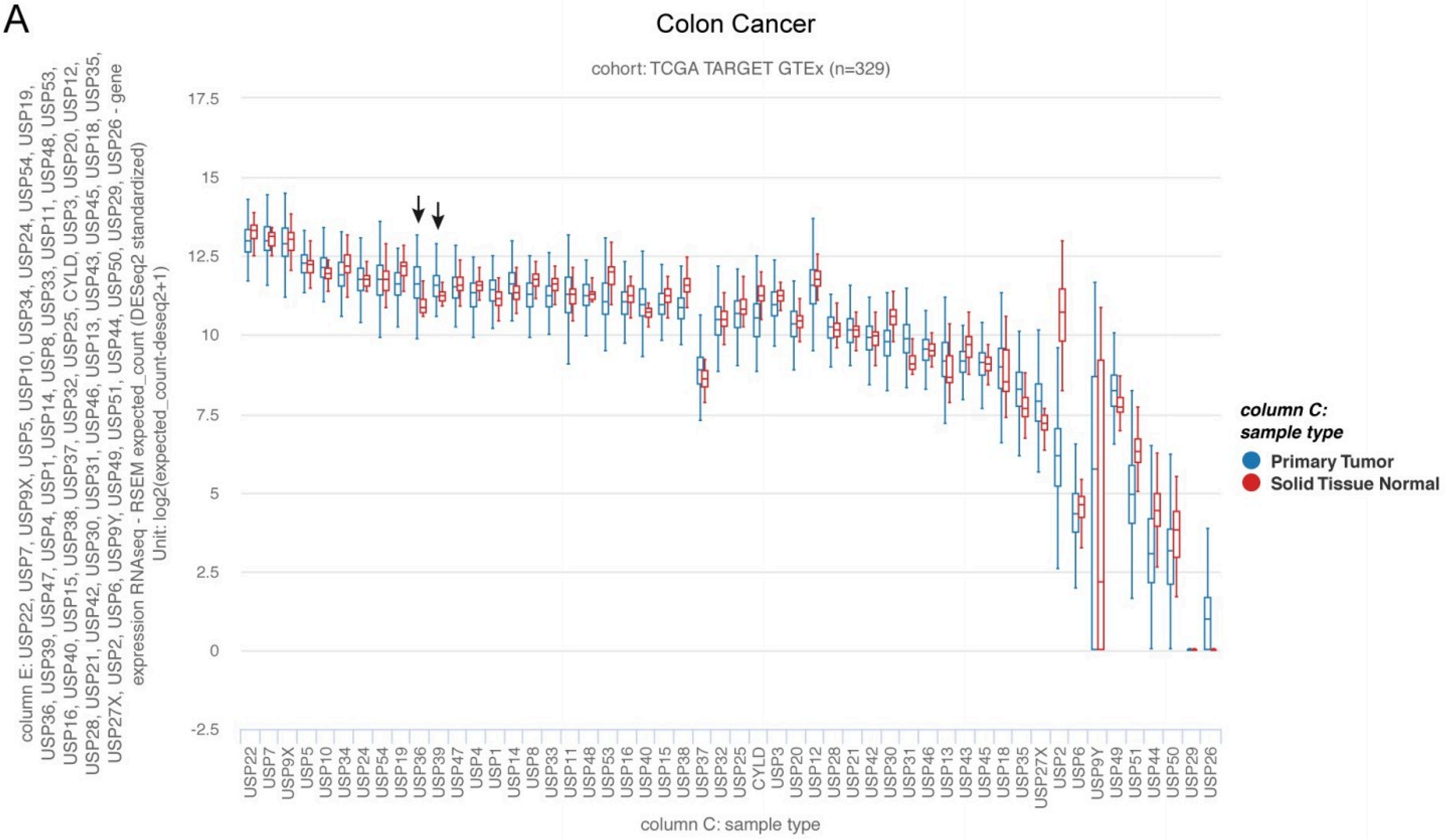

B

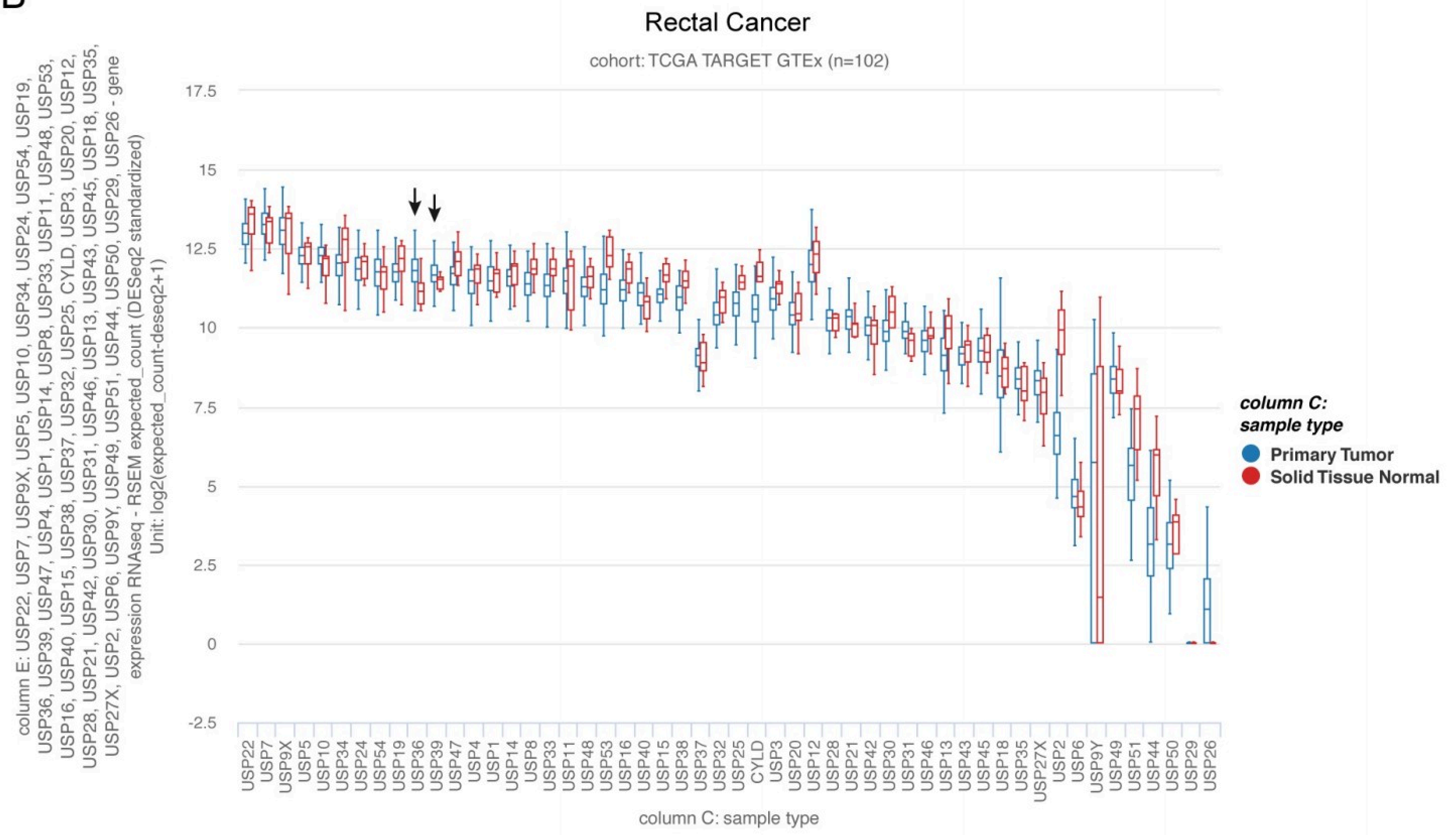

Figure S1. USPs expression compared between tumors and normal tissues of patients with colorectal cancer.
(A) USPs expression levels in COAD tumor versus normal samples in TCGA are shown in the histogram. USP36 and USP39 were marked.
(B) USPs expression levels in READ tumor versus normal samples in TCGA are shown in the histogram. USP36 and USP39 were marked.

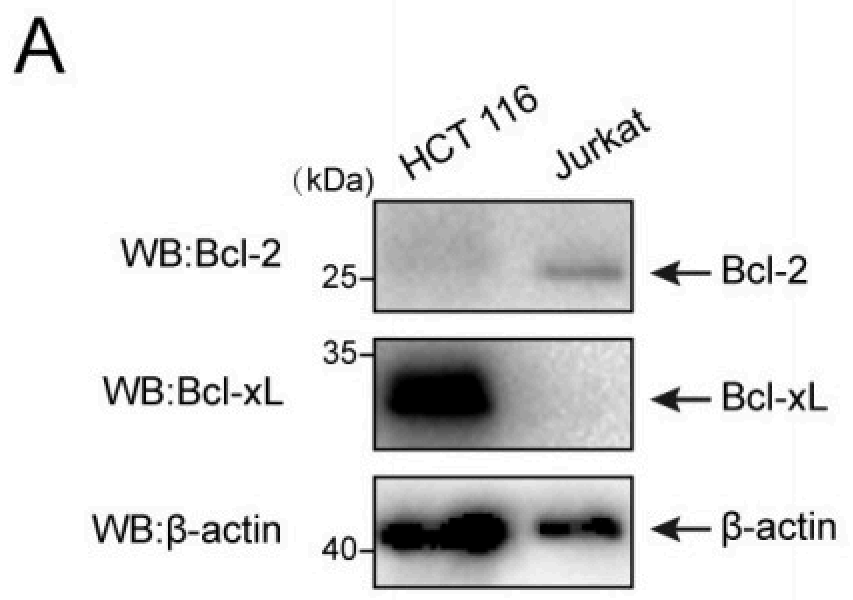

Figure S2. Expression pattern of Bcl-2 family members in solid tumor and hematological cancer cell lines.
(A) Proteins from HCT 116 and Jurkat cells were extracted and blotted with indicated antibodies.

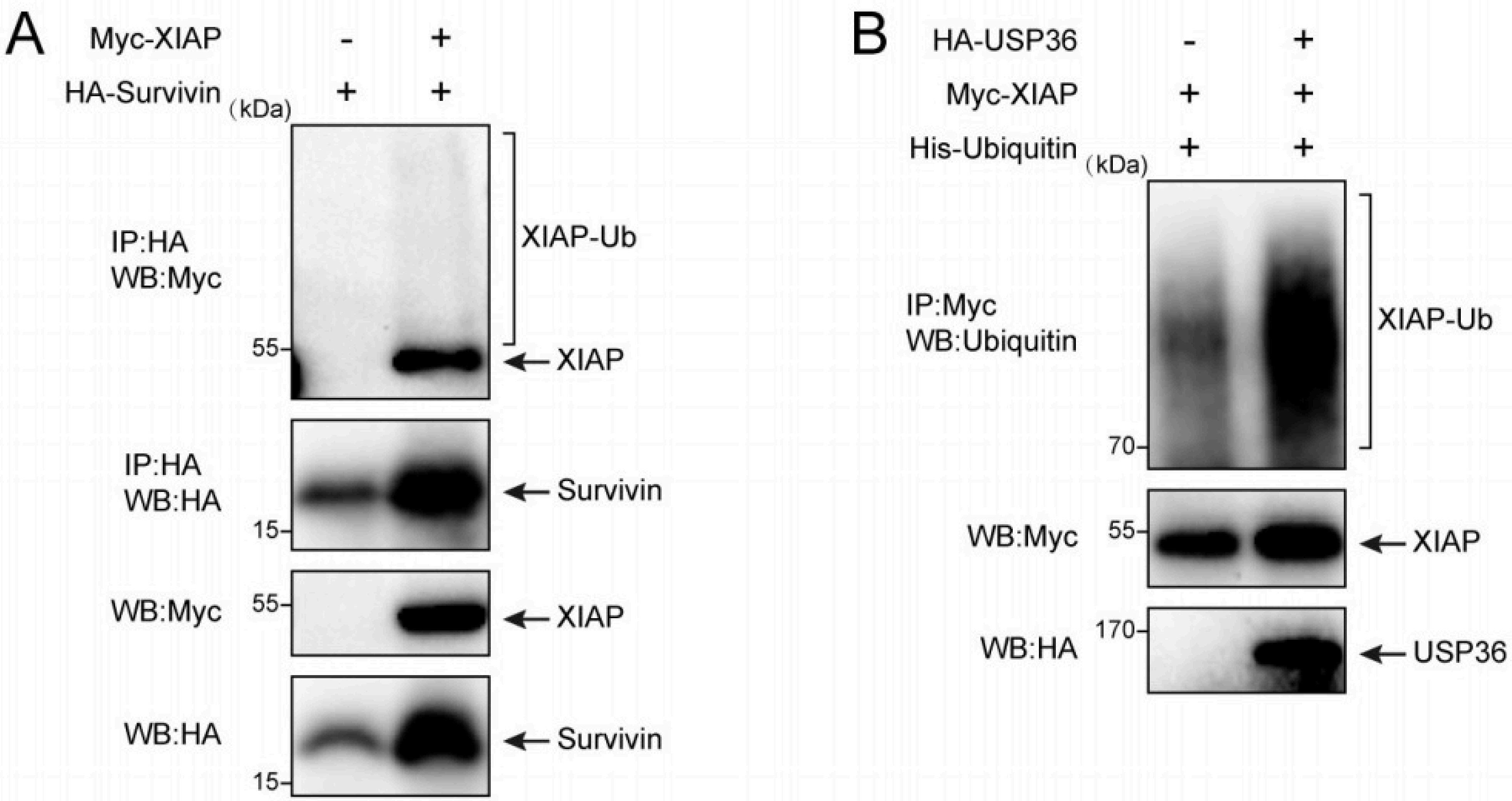

Figure S3. Survivin and USP36 elevated ubiquitination levels of XIAP

(A) HEK 293 cells were transfected with HA-survivin alone, or together with Myc-XIAP, immunoprecipitated with anti-HA antibody and analyzed with indicated antibodies.

(B) HEK 293 cells were transfected with XIAP and His-ubiquitin, or together with HA-USP36. XIAP was immunoprecipitated with Myc-antibody, then ubiquitination levels were analyzed by anti-ubiquitin antibody..
